# Headache: A striking prodromal and persistent symptom, predictive of COVID-19 clinical evolution

**DOI:** 10.1177/0333102420965157

**Published:** 2020-11-04

**Authors:** Edoardo Caronna, Alejandro Ballvé, Arnau Llauradó, Victor José Gallardo, Diana María Ariton, Sofia Lallana, Samuel López Maza, Marta Olivé Gadea, Laura Quibus, Juan Luis Restrepo, Marc Rodrigo-Gisbert, Andreu Vilaseca, Manuel Hernandez Gonzalez, Monica Martinez Gallo, Alicia Alpuente, Marta Torres-Ferrus, Ricard Pujol Borrell, José Alvarez-Sabin, Patricia Pozo-Rosich

**Affiliations:** 1 Neurology Department, Hospital Universitari Vall d’Hebron, Department of Medicine, Universitat Autònoma de Barcelona, Barcelona, Spain; 2 Headache and Neurological Pain Research Group, Vall d’Hebron Research Institute, Department of Medicine, Universitat Autònoma de Barcelona, Barcelona, Spain; 3 Immunology Department, Hospital Universitari Vall d’Hebron, Vall d’Hebron Research Institute, Barcelona, Spain

**Keywords:** Headache, COVID-19, SARS-CoV-2, prognosis, loss of smell

## Abstract

**Objective:**

To define headache characteristics and evolution in relation to COVID-19 and its inflammatory response.

**Methods:**

This is a prospective study, comparing clinical data and inflammatory biomarkers of COVID-19 patients with and without headache, recruited at the Emergency Room. We compared baseline with 6-week follow-up to evaluate disease evolution.

**Results:**

Of 130 patients, 74.6% (97/130) had headache. In all, 24.7% (24/97) of patients had severe pain with migraine-like features. Patients with headache had more anosmia/ageusia (54.6% vs. 18.2%; *p* < 0.0001). Clinical duration of COVID-19 was shorter in the headache group (23.9 ± 11.6 vs. 31.2 ± 12.0 days; *p* = 0.028). In the headache group, IL-6 levels were lower at the ER (22.9 (57.5) vs. 57.0 (78.6) pg/mL; *p* = 0.036) and more stable during hospitalisation. After 6 weeks, of 74 followed-up patients with headache, 37.8% (28/74) had ongoing headache. Of these, 50% (14/28) had no previous headache history. Headache was the prodromal symptom of COVID-19 in 21.4% (6/28) of patients with persistent headache (*p* = 0.010).

**Conclusions:**

Headache associated with COVID-19 is a frequent symptom, predictive of a shorter COVID-19 clinical course. Disabling headache can persist after COVID-19 resolution. Pathophysiologically, its migraine-like features may reflect an activation of the trigeminovascular system by inflammation or direct involvement of SARS-CoV-2, a hypothesis supported by concomitant anosmia.

## Introduction

The global pandemic caused by the Severe Acute Respiratory Syndrome-Coronavirus-2 (SARS-CoV-2) is putting our healthcare systems at stake. COVID-19 is the infectious disease caused by this viral pathogen, and which can lead, in some patients, to respiratory failure amongst other severe symptoms, requiring urgent medical aid. In Spain, COVID-19 had been diagnosed in as many as 200,210 people by 19 April 2020 (1), forcing hospitals to reorganise in a profound, quick way. Specifically, neurologists have been attending COVID-19 patients at the emergency room (ER), which has enabled them to observe the presence of neurological symptoms associated with COVID-19 otherwise not analysed in detail. At the ER, severe, difficult-to-treat headache, together with anosmia and ageusia, has been a major initial neurological complaint. Despite the fact that the Centres for Disease Control and Prevention (2) include headache as one of the main symptoms of COVID-19, a better definition of headache itself is lacking and no data on its evolution are available at present. Moreover, it is unknown whether headache may represent a relevant clinical symptom, predicting the course of COVID-19 itself, a fact that could guide clinicians in their evaluations of COVID-19 patients in future waves of the pandemic.

“Headache attributed to systemic viral infection” is included in the International Classiﬁcation of Headache Disorders third edition (ICHD-3) (3) and, although commonly reported (4), specific data are lacking. COVID-19 gives the opportunity to define its headache characteristics, which until now have mainly been described in terms of prevalence, with discrepant results, the prevalence in studies based on clinical charts review in China (8–23%) (5–8) or Spain (14.1%) (9) being substantially different from interview-based cross-sectional study (70%) (10).

As studies related to headache associated with viral infections are lacking, and COVID-19 is clearly associated with headache, we decided to define and describe headache characteristics and evolution in relation to COVID-19, focusing on it as a possible prognostic factor. Moreover, we decided to correlate the presence of headache with systemic inflammatory responses and hypothesise about its pathophysiological mechanisms.

## Methods

This is a prospective study conducted in a Spanish tertiary hospital in Barcelona (Vall d’Hebron Hospital).

During the COVID-19 pandemic, neurologists started working 24/7 at the ER as general clinicians. They recruited, during a 3-week period (28 March to 22 April 2020), all the consecutive patients with COVID-19 symptoms attended by them (ER patients were assigned randomly by triage to clinicians to be visited), including only those who could give consent and undergo a full interview. COVID-19 symptoms were pre-specified for all neurologists and were based on the list of symptoms reported by the World Health Organization (11). We collected demographic data, COVID-19 symptoms, family and personal history of any headache (ICHD-3) (3), categorising patients as episodic or chronic. If patients had experienced headache at any time during COVID-19, we collected the date of onset and cessation in relation to other COVID-19 symptoms as well as headache characteristics. Headache pain severity was defined as mild if patients considered that, in the absence of other COVID-19 symptoms, headache alone would allow them to carry on with their daily activities as usual; moderate if they had to reduce their daily activities, and severe if they had to stop doing any kind of task.

In our hospital, according to the COVID-19 ER protocol, we recorded vital signs and performed a physical examination and a chest X-ray to rule out pneumonia. At the ER, patients with COVID-19 symptoms but with normal vital signs, negative X-ray and normal physical examination could be immediately discharged without undergoing nasopharyngeal swabs to confirm SARS-CoV-2 infection. In all other cases, patients were admitted for further testing and/or treatment, including a real-time reverse transcriptase polymerase -chain reaction (RT-PCR) assay by nasopharyngeal swabs to confirm SARS-Cov-2 and blood testing with inflammatory markers (C-reactive protein–CRP: 0.03–0.50 mg/dL; interleukin-6-IL-6: 0–4.3 pg/mL; ferritin: 25–250 ng/mL; Lactate Dehydrogenase–LDH: 0–248 UI/L; D-dimer: 0–243 ng/mL). In hospitalised patients, we recorded treatment administered, presence of other medical complications, hospital and intensive care unit (ICU) length of stay and mortality, by periodically revising electronic medical charts. Then, we analysed all data, described the cohort of patients with headache associated with COVID-19, and compared them with those not having headache.

After 6 weeks from admission, we followed up patients by phone call to evaluate persistence of headache and its characteristics as well as other COVID-19 symptoms through a structured survey. Then, we compared baseline and post-6-week data.

To define prognosis in our cohort, we used these variables: COVID-19 disease duration, defined as the number of days between the onset of the first and the resolution of the last COVID-19 symptom, hospital length of stay, and all-cause in-hospital mortality.

### Statistical analysis

We obtained descriptive and frequency statistics and made comparisons using the SPSS, version 21.0 for Windows. We reported nominal (categorical) variables as frequencies (percentages), and continuous variables as mean ± standard deviation (age, temperature, CRP and LDH) or median and interquartile range (IQR) (IL-6, D-dimer and ferritin). We checked the normality assumption of quantitative variables through visual methods (Q-Q plots) and normality tests (Kolmogorov-Smirnov test). We assessed statistical significance for intergroup variables by Pearson’s chi-square when comparing categorical variables. In the case of having an expected count of less than 5 in more than 20% of cells in the contingency table, we used Fisher’s exact test. We used linear trend chi-square for ordinal variables, independent t-test for continuous variables that followed a normal distribution and the Mann-Whitney U test for the rest of the continuous variables. In order to evaluate the COVID-19 prognosis (COVID-19 disease duration, hospital length of stay and mortality) at the follow-up, we used one-way analysis of covariance (ANCOVA), adjusted for the effect of age and gender, for the group with and without headache. The false discovery rate (FDR) with Benjamini-Hochberg procedure was used to correct *p*-values for multiple comparisons.

Finally, we studied the role of inflammatory biomarkers and fever (≥37.5°C) in a subgroup of patients with ongoing headache at the ER and an equally age/gender matched group without headache in order to avoid biased results. We also conducted a longitudinal data analysis in order to model the inflammatory biomarker changes over the course of COVID-19 between patients with and without headache. We only selected hospitalised patients with at least three available blood tests that had been done at the same timepoints in the course of COVID-19 disease, starting from the onset of their COVID-19 symptoms. Longitudinal analysis was performed using linear mixed-effects models fitted by maximum likelihood and adjusted by age. Models were computed using the nlme (v3.1-144) package from R.

We did not conduct a statistical power calculation prior to the study because the sample size was based on the available data. Missing values were imputed using the MICE (Multivariate Imputation via Chained Equations) package from R (v3.8.0) (12). Concerning missing values, there was <5% of missingness in nominal variables (headache localisation, quality of pain, and pain severity). Hence, we used a Bayesian polytomous regression as a method of imputation for headache localisation and quality of pain and a proportional odds model for headache pain severity. The missingness in continuous variables (temperature, CRP, IL-6, D-dimer, ferritin, LDH) was rated between 2% (temperature) to 18% (LDH). In that case, we used random forest imputations in order to estimate these values according to their other variables.

*p*-values presented are for a two-tailed test and we considered *p*-values < 0.05 statistically significant.

### Ethics approval and patients’ consent

The study was approved by the Vall d’Hebron Ethics Committee (PR(AG)227/2020). All patients gave written informed consent for the analysis of patients’ data which was collected according to Spanish regulation on clinical trials.

## Results

We included 130 adult patients. Table 1 shows their characteristics and all COVID-19 symptoms reported at ER. From the 130 patients included, 74.6% (97/130) had experienced headache as a COVID-19 symptom, while the other 33 did not.

**Table 1.** Clinical characteristics of COVID-19 patients at ER.

Demographic characteristics (n = 130)	
Sex, n (%)	
Male	64 (49.2)
Female	66 (50.8)
Age (years), n (%)	
Mean (SD)	53.9 (16.4)
<34	16 (12.3)
35–44	23 (17.7)
45–54	30 (23.1)
55–64	25 (19.2)
≥65	36 (27.7)
COVID-19 characteristics	
Reported symptoms at ER, n (%)	
Headache	97 (74.6)
Fever	115 (88.5)
Malaise	60 (46.2)
Myalgia	39 (30.0)
Dizziness	19 (14.6)
Cough	105 (80.2)
Dyspnea	81 (62.3)
Chest pain	4 (3.0)
Expectoration	19 (14.6)
Odynophagia	12 (9.2)
Loss of smell/taste	59 (45.4)
Diarrhea	36 (27.7)
Radiological findings at ER, n (%)	
Pneumonia	103 (79.2)
Bilateral pneumonia	77 (59.2)
COVID-19 Confirmation (positive RT-PCR), n (%)	89 (68.5)
Hospitalisation, n (%)	104 (80.0)
Vital signs and inflammatory markers at the ER	
O_2_ requirements, n (%)	31 (23.8)
Fever, n (%)	45 (34.6)
Lymphopenia, n (%)	70 (53.8)
CRP, mean ± SD, mg/ml	9.3 ± 8.4
IL-6, median (IQR), pg/ml	34.0 (62.1)
D-dimer, median (IQR), ng/ml	231.0 (243)
Ferritin, median (IQR), ng/ml	354.0 (406)
LDH, mean ± SD, UI/L	369.4 ± 221.4

ER: Emergency room; SD: standard deviation; IQR: interquartile range; ICU: intensive care unit; RT-PCR: real-time reverse transcriptase polymerase-chain reaction; O_2_: oxygen; lymphopenia (<1.0 × 10^9^/L); CRP: C-reactive protein; IL-6: interleukin-6; LDH: lactate dehydrogenase.

### Headache and COVID-19 at ER

**Figure 1.**
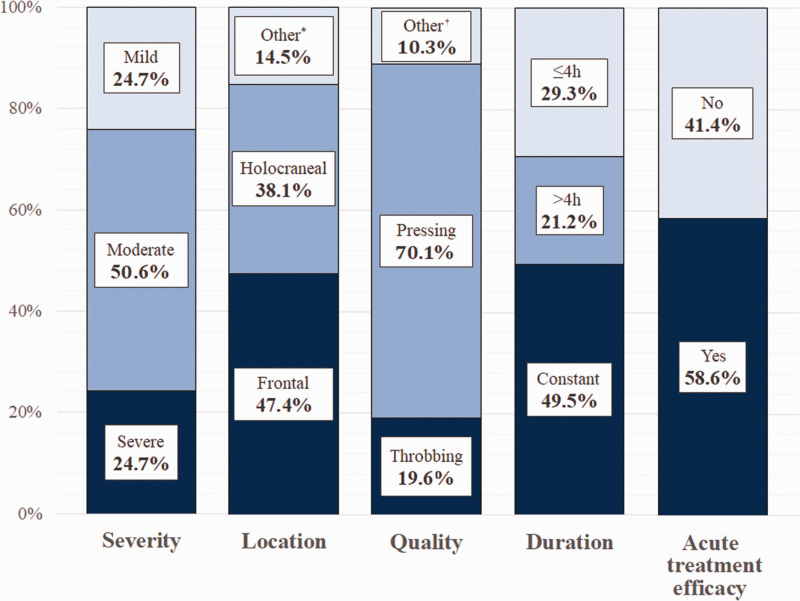
Headache associated with COVID-19 characteristics. *Other headache locations reported were frontocervical (6.2%), hemicranial (5.2%) and cervical (4.1%). †Other reported pain qualities were drilling (5.2%), shooting (4.1%) and burning (1.0%).

In our cohort of headache patients, 57.7% (56/97) were female, the mean age was 50.6 ± 15.3 years old and 19.6% (19/97) had a personal history of episodic migraine. No patients had a history of chronic migraine. Headache-associated symptoms reported by patients were nausea and vomiting (25/97), worsening with movement (12/97) photo/phonophobia (10/97), vertigo (4/97) and subjective neck stiffness (3/97). Specific headache features are reported in Figure 1.

At the ER, the neurological examination together with symptoms evaluation, performed by neurologists, ruled out meningitis in all recruited patients with headache. Based on the striking clinical observation of some patients with severe headache at ER, we compared patients with severe headache (24/97) with the ones with mild-moderate pain (73/97), specifically analysing migraine-like features (Table 2).

**Table 2.** Comparison between patients with severe and mild-moderate headache at ER (n = 97).

	Severe headache (n = 24)	Mild-moderate headache (n = 73)	Adjusted *p-*value^a^
Demographic characteristics			
Age (years), mean ± SD	44.8 ± 14.9	52.5 ± 15.1	0.049*
Sex (female), n (%)	20 (83.3)	36 (49.3)	0.004**
History of any type of headache, n (%)	10 (41.7)	21 (28.8)	0.313
History of migraine, n (%)	6 (25.0)	13 (17.8)	0.554
Headache characteristics			
Onset before another COVID-19 symptom, n (%)	6 (25.0)	3 (4.1)	0.007**
Time since headache onset to ER presentation (days), median (IQR)	10.5 (10.0)	6.0 (6.0)	0.002**
Holocraneal pain, n (%)	9 (37.5)	28 (38.4)	1.000
Pain quality, n (%)			
Pressing	11 (45.8)	57 (78.1)	0.004**
Throbbing	9 (37.5)	10 (13.7)	0.017*
Other migraine features, n (%)			
Worsening with movement	6 (25.0)	6 (8.2)	0.066
Nausea and vomiting	11 (45.8)	14 (19.2)	0.015*
Photo/phonophobia	5 (20.8)	5 (6.8)	0.114
Daily constant pain, n (%)	18 (75.0)	30 (41.1)	0.005**
Response to acute treatment, n (%)	9 (37.5)	48 (65.8)	0.018*

^a^Adjusted *p*-value with Benjamini-Hochberg procedure.

*p-value ≤0.05.

**p-value ≤0.01.

ER: Emergency room; SD: standard deviation; IQR: interquartile range.

Then, comparing patients with or without headache, we observed that the first group were younger (50.6 ± 15.3 vs. 63.6 ± 15.7; *p* < 0.0001), there were more females (57.7% vs. 30.3%; *p* = 0.009) and reported higher headache history of any type (32.0% vs. 12.1%; *p* = 0.039). In regards to COVID-19 symptoms, the most relevant result was that in the headache group more patients had anosmia/ageusia (54.6% vs. 18.2%; *p* < 0.0001). All other variables are shown in Table 3.

**Table 3.** Comparison between COVID-19 patients with and without headache at the ER.

	No headache (n = 33)	Headache (n = 97)	Adjusted *p*-value^a^
Demographic characteristics			
Age (years), mean ± SD	63.6 ± 15.7	50.6 ± 15.3	<0.0001**
Sex (female), n (%)	10 (30.3)	56 (57.7)	0.009**
History of any type of headache, n (%)	4 (12.1)	31 (32.0)	0.039*
History of migraine, n (%)	2 (6.1)	19 (19.6)	0.099
COVID-19 characteristics			
Reported symptoms at ER, n (%)			
Fever	28 (84.8)	87 (89.7)	0.529
Malaise	8 (24.2)	52 (53.6)	0.004**
Myalgia	7 (21.2)	32 (33.0)	0.272
Dizziness	1 (3.0)	18 (18.6)	0.042*
Cough	24 (72.7)	81 (83.5)	0.204
Dyspnea	21 (63.6)	60 (61.9)	1.000
Chest pain	2 (6.1)	1 (1.0)	0.158
Expectoration	1 (3.0)	18 (18.6)	0.042*
Odynophagia	1 (3.0)	11 (11.3)	0.294
Loss of smell/taste	6 (18.2)	53 (54.6)	<0.0001**
Diarrhea	8 (24.2)	28 (28.9)	0.660
Pneumonia	25 (75.8)	78 (80.4)	0.622
Bilateral pneumonia	21 (63.6)	56 (57.7)	0.682
COVID-19 confirmation (RT-PCR), n (%)	23 (69.7)	66 (68.0)	1.000
Hospitalisation, n (%)	27 (81.8)	77 (79.4)	1.000

^a^Adjusted *p*-value with Benjamini-Hochberg procedure.

*p-value ≤0.05.

**p-value ≤0.01.

ER: Emergency room; RT-PCR: real-time reverse transcriptase polymerase-chain reaction.

### COVID-19 evolution: Headache as a prognostic factor

Of 130 patients, 80.0% (104/130) were hospitalised after ER evaluation. In all hospitalised patients, we could follow their clinical course by electronic chart and observed that 8.5% (11/130) required ICU. Mortality was 3.1% (4/130, one patient belonging to the headache group and three to the group without headache). We did not detect new onset headaches through chart review during hospitalisation.

After 6 weeks, we could get in touch with 100 patients of our cohort (74 with headache and 26 without headache) and interviewed them about disease evolution. There were no statistically significant differences with regard to the demographic variables in patients that were not followed up.

From these, 27.0% (27/100) was still experiencing at least one symptom of COVID-19 other than headache. Those without any more symptoms of COVID-19 had a mean duration of disease of 25.8 ± 11.9 days.

Interestingly, comparing patients with and without headache, for whom data were available at follow-up, and adjusting for age and gender, we observed shorter COVID-19 disease duration in the headache group (23.9 ± 11.6 vs. 31.2 ± 12.0 days; *p* = 0.028). We did not observe any difference in mortality (no mortality in this subgroup) or hospital length of stay (9.1 ± 9.0 vs. 10.9 ± 9.0 days; *p* = 0.854).

### Headache evolution

After 6 weeks, of the 74 headache patients, 37.8% (28/74) still had headache. Those patients whose headache had stopped had a mean duration of the symptom of 15.4 ± 11.1 days. Then, we analysed patients with ongoing headache after 6 weeks, observing that 50% of them (14/28) had never suffered from recurrent headache before. A total of 60.7% of patients (17/28) had daily constant headache. Response to acute treatment was insufficient both at baseline and follow-up, without statistically significant differences at the two timepoints (32.1% vs. 28.6%; *p* = 0.701).

Then, we compared patients with ongoing headache after 6 weeks with those who were headache free. Significant variables associated with persisting headache are shown in Table 4.

**Table 4.** Comparison between patients with ongoing headache and with headache resolution during follow-up (n = 76).

	Ongoing headache (n = 28)	Headache resolution (n = 46)	Adjusted *p-*value^a^
Demographic characteristics			
Age (years), mean ± SD	49.2 ± 15.7	52.5 ± 15.7	0.386
Sex (female), n (%)	23 (82.1)	22 (47.8)	0.004**
History of any type of headache, n (%)	14 (50.0)	12 (26.1)	0.047*
History of migraine, n (%)	8 (28.6)	8 (17.4)	0.383
COVID-19 characteristics			
Reported symptoms at ER, n (%)			
Fever	25 (89.3)	41 (89.1)	1.000
Malaise	15 (53.6)	26 (56.5)	0.815
Myalgia	12 (42.9)	14 (30.4)	0.321
Dizziness	9 (19.6)	5 (17.9)	1.000
Cough	22 (78.6)	40 (87.0)	0.352
Dyspnea	22 (78.6)	26 (56.5)	0.079
Chest pain	1 (3.6)	0 (0.0)	0.717
Expectoration	3 (10.7)	7 (15.2)	0.733
Odynophagia	4 (14.3)	3 (6.5)	0.415
Loss of smell/taste	16 (57.1)	29 (63.0)	0.632
Diarrhea	5 (17.9)	17 (37.0)	0.116
Pneumonia	39 (84.8)	21 (75.0)	0.364
Bilateral pneumonia	28 (60.9)	15 (53.6)	0.629
Persistent symptoms at follow-up, n (%)	19 (67.9)	9 (10.6)	<0.001**
COVID-19 disease duration (days), Median (IQR)	26.5 (21.5)	23.0 (12.5)	0.126
Hospitalisation, n (%)	21 (75.0)	39 (89.4)	0.364
Days of hospitalisation, median (IQR)	6.0 (13.5)	5.5 (7.5)	0.971
ICU, n (%)	4 (14.3)	4 (8.7)	0.467
Headache characteristics			
Onset before another COVID-19 symptom, n (%)	6 (21.4)	2 (4.4)	0.010**
Holocraneal pain, n (%)	10 (35.7)	18 (39.1)	0.809
Pain quality, n (%)			
Pressing	19 (67.9)	33 (71.7)	0.796
Throbbing	5 (17.9)	9 (19.6)	1.000
Moderate-severe pain, n (%)	25 (89.3)	32 (69.6)	0.085
Daily constant pain, n (%)	17 (60.7)	22 (47.8)	0.341
Headache associated symptoms at ER, n (%)			
Nausea and vomiting	9 (32.1)	12 (26.1)	0.604
Photo/phonophobia	1 (3.6)	6 (8.1)	0.242
Vertigo	1 (3.6)	2 (4.4)	1.000
Neck stiffness	1 (3.6)	1 (2.2)	1.000
Worsening with movement	6 (21.4)	6 (13.0)	0.352
Response to acute treatment, n (%)	9 (32.1)	34 (73.9)	0.001**

^a^Adjusted *p*-value with Benjamini-Hochberg procedure.

*p-value ≤0.05.

**p-value ≤0.01.

ER: Emergency room; SD: standard deviation; IQR: interquartile range; ICU: intensive care unit.

### Headache related to inflammation and fever

We selected patients with ongoing headache at the ER, when vital signs and blood samples were collected, and an equally age/gender-matched group without headache. We included 60 patients, 36 with headache and 24 without. We observed statistically significantly lower levels of IL-6 and LDH. We found no differences in presence of fever at the ER between the two groups. Other variables are shown in Table 5.

**Table 5.** Comparison in inflammatory biomarkers between age/gender-matched COVID-19 patients with and without headache.

	Headache (n = 36)	No headache (n = 24)	Adjusted *p*-value^a^
Demographic characteristics			
Age (years), mean ± SD	59.1 ±14.2	61.1 ± 14.9	0.594
Sex (female), n (%)	21 (58.3)	15 (41.7)	0.280
COVID-19 characteristics			
COVID-19 confirmation (RT-PCR), n (%)	25 (69.4)	15 (62.5)	0.708
COVID-19 disease duration at ER, days, median (IQR)	8.5 (7.5)	9.0 (9.8)	0.623
Vital signs and inflammatory biomarkers			
Fever, n (%)	11 (30.6)	4 (16.7)	0.482
Lymphopenia, n (%)	20 (55.6)	16 (66.7)	0.432
CRP, mean ± SD, mg/ml	8.9 ± 7.9	11.7 ± 9.8	0.381
IL-6, median (IQR), pg/ml	22.9 (57.5)	57.0 (78.6)	0.036*
D-dimer, median (IQR), ng/ml	300.0 (3300)	250.0 (1593.0)	0.481
Ferritin, median (IQR), ng/ml	488.0 (466.0)	287.0 (110.0)	0.052
LDH, mean ± SD, UI/L	302.8 ± 107.7	457.1 ± 207.6	0.016*

^a^Benjamini-Hochberg adjusted *p*-value with a false discovery rate greater than 0.05.

*p value ≤0.05.

**p value ≤0.01.

SD: standard deviation; RT-PCR: real-time reverse transcriptase polymerase-chain reaction; ER: emergency room; IQR: interquartile range;CRP: C-reactive protein; IL-6: interleukin-6; LDH: lactate dehydrogenase.

**Figure 2.**
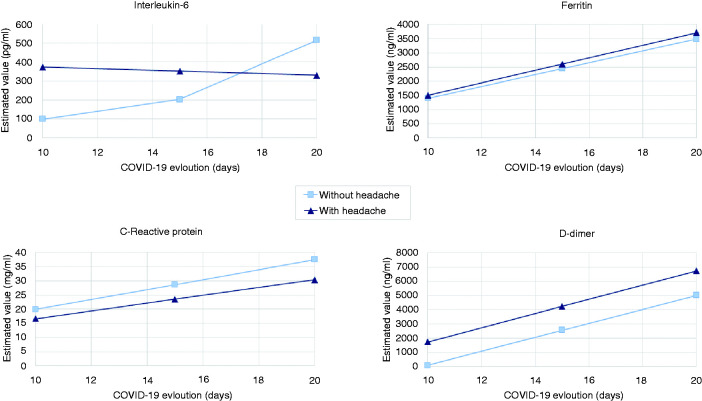
Evolution of inflammatory biomarkers (IL-6, CRP, ferritin and D-dimer) during the progression of COVID-19 disease. Once having analysed mean values of inflammatory biomarkers at the ER (baseline), we decided to exclusively evaluate the trends of their evolution over time. Of 24 hospitalised patients in whom IL-6, CRP, ferritin and D-dimer data were available at the same three points in time, 18 had headache while six did not. There were no differences in time passed between each blood test and COVID-19 onset. Only IL-6 changes over COVID-19 progression were significant when comparing patients with and without headache (*p* = 0.003) in the linear mixed-effects models adjusted by age.

To specifically analyse the evolution of inflammatory biomarkers over time, we included 24 patients in this sub-analysis: 18 had headache, while six did not. There were no statistically significant differences either in patients’ age (headache: 56.6 ± 9.8 vs. no-headache: 63.3 ± 6.7 years; *p* = 0.130) or in gender (female – headache: 55.6% vs. no-headache: 66.7%; *p* = 1.000). Only IL-6 significantly changed over time between the two groups (*p* = 0.003), observing more stable levels of IL-6 during COVID-19 in patients with headache (Figure 2).

## Discussion

In March 2020, during the COVID-19 pandemic, we noticed that some patients were complaining of headache while others were not, which made us wonder why.

Our study aimed to first describe the characteristics and evolution of headache associated with COVID-19, to correlate it with the evolution of the disease, and generate a hypothesis on the underlying pathophysiological mechanisms, with the final goal of helping clinicians to better understand this symptom and its clinical relevance.

### Clinical presentation of headache associated with COVID-19

Headache attributed to COVID-19 is common in both genders and middle-aged people, while patients without headache are older and male. History of headache of any type is more common in patients presenting headache as a COVID-19 symptom, but not exclusive. Interestingly, the presence of headache in COVID-19 is significantly associated with other cranial symptoms, anosmia/ageusia. Specifically, these symptoms have been frequently reported in case reports of patients with headache during COVID-19 (13–14).

Concerning headache characteristics in COVID-19, a recent study conducted in Spain (15) has detected different phenotypes on the basis of headache features and the presence of personal history of primary headaches. In our cohort, we observed that in the majority of patients headache is mild/moderate, while one fourth, especially women and younger subjects, have a severe “migraine-like” headache that is not associated with a more personal migraine history. Although headache attributed to COVID-19 usually starts with the other COVID-19 symptoms, patients with severe pain more often have it as a prodromal symptom. Headache as the first COVID-19 symptom has been described in a recent case report (16).

Concerning headache evolution, surprisingly we observed that one third of followed-up patients had persistent disabling daily headache after 6 weeks, with poor response to acute treatment and, in more than 30%, representing the only symptom left of COVID-19. Interestingly, up to 50% of these patients had no history of recurrent headache at all, a fact that could lead to the onset of a “new daily” headache. Moreover, in a relevant number of these cases, headache was an initial prodromal symptom of COVID-19. In our study, headache was more likely to persist in females and patients with a headache history, although no patients had a chronic primary headache before COVID-19.

### Relevance of assessing headache in COVID-19 for prognosis

As of 13 May 2020, the seroprevalence of antibodies against COVID-19 in the Spanish population is only 5%, making SARS-CoV-2 an ongoing health threat in our country (17). In our study, we observed that, among patients’ reported COVID-19 symptoms, only the presence of headache is predictive of better prognosis, in terms of an almost 1-week shorter COVID-19 disease. Although recent studies (18–19) have shown that anosmia could be useful in detecting COVID-19 cases, more specifically pointing to SARS-CoV-2 infection, we remark that headache represents a relevant symptom of COVID-19 in terms of prognosis, raising awareness of the importance of its assessment.

### Hypothesis on underlying pathophysiological mechanisms

Usually patients and clinicians blame fever as the cause of headache associated with viral infections. However, its role in causing headache is still a matter of debate and possible mechanisms involve increase of proinflammatory cytokines (20). In a recent study on COVID-19, headache seemed to present independently from fever (15), in line with our findings observing that headache was not associated with presence of fever at the ER or as a reported symptom. Although our study was not specifically designed to address the relationship between fever and headache, our findings support the need to further evaluate whether fever has a minor role in directly causing headache, being more directly related to the initial response to the pathogen.

Concerning specific pathophysiological mechanisms, it has been hypothesised that SARS-CoV-2 may trigger a hyperinflammatory state in some patients (cytokine storm) (21) especially through IL-6 (22–23), whose levels seem to correlate with dysregulation of other coagulation and inflammatory biomarkers in a recent proteomic study (24). The role of IL-6 has been also demonstrated in neuroinflammation (25) and specifically in migraine (26–27). Therefore, it is logical to wonder whether the suggested inflammatory state observed in COVID-19 is also responsible for neuroinflammation, leading to headache. We observed lower levels of IL-6 at the ER in the headache group, a finding that cannot be explained by different stages of the disease between groups. This fact is surprising, considering that COVID-19 patients with headache had more personal headache history. Moreover, we observed that during COVID-19 evolution, levels of IL-6 seemed to be more stable in patients with headache compared to the ones without it. Considering that headache patients also had shorter COVID-19 disease evolution, their lower and more stable IL-6 levels may indicate that inflammation may be kept at a more localised level.

In this context, considering that headache attributed to COVID-19 could mimic migraine even in individuals without a personal history of this condition, we hypothesise that there is a meningeal peripheral sensitisation and therefore an activation of the trigeminovascular system (28) underlying this headache type.

Amongst possible mechanisms that could lead to the activation of the trigeminovascular system, it is worth mentioning our finding on the association between headache and anosmia/ageusia as distinctive symptoms of COVID-19 (5,29). It has been hypothesised that SARS-CoV-2 has neurotropic characteristics, being able to invade peripheral nerve terminals and enter the central nervous system through trans-synaptic pathways (30–32). Inside the nasal cavity, concomitant anosmia may indicate a peripheral activation of the trigeminovascular system mediated by the pathogen itself, acting not only on the specialised olfactory epithelium, but also on trigeminal branches present at this level or through the olfactory-trigeminal interactions. As for other pathophysiological mechanisms, endothelitis may also have a relevant role in COVID-19 (33), since inflammatory reactions could be generated through the interaction between the virus and its receptor, angiotensin-converting enzyme 2 (ACE2), which is present in the endothelium of blood vessels. ACE2 is expressed in the endothelium of small vessels of cerebral circulation (34), that SARS-CoV-2 can reach through bloodstream dissemination (35). Thus, the presence of ACE2 in the meningeal endothelium, leading to trigeminovascular sensitisation, should be further investigated as a potential pathophysiological mechanism for COVID-19 headache.

**Figure 3.**
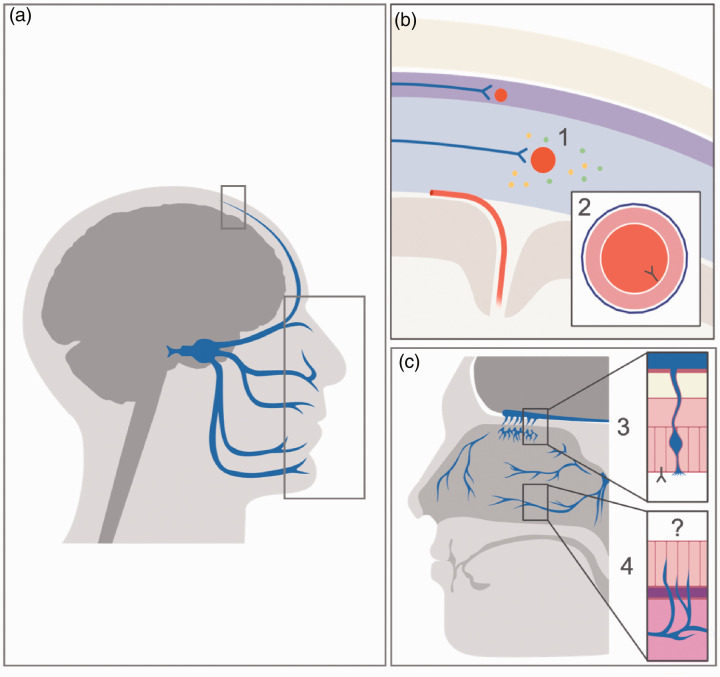
Possible pathophysiological mechanisms of headache associated with COVID-19. (a) Head section representing trigeminal innervation, including meninges and nasal cavities (selected areas that are enlarged in (b) and (c). (b) At meningeal level, the trigeminal afferents (blue arrows) innervate meningeal vessels (represented in red), creating the trigeminovascular system. Its activation may be due to i) systemic inflammation that may facilitate meningeal sensitisation leading to a local release of inflammatory peptides that stimulates trigeminal terminals; ii) direct binding of SARS-CoV-2 from the bloodstream on ACE2, which is expressed by the endothelium of meningeal vessels, causing endothelitis and therefore inflammation. (c) In the nasal cavities, both the specialised olfactory epithelium and the nasal epithelium are present, the latter being innervated by trigeminal nerve afferents. iii) The supporting cells of the olfactory epithelium, in which the olfactory neurons are embedded, express ACE2, where the SARSCoV- 2 may bind, causing anosmia, a symptom that is significantly associated with presence of headache. iv) At the level of the nasal epithelium, the trigeminal system may be peripherally activated by the direct action of SARS-CoV-2 on the nasal epithelium or the trigeminal branches, or by an indirect pathway involving the interactions between the olfactory and trigeminal innervation. These mechanisms should be further studied.

All the previously mentioned pathophysiological mechanisms are shown in Figure 3.

It could be interesting for future studies to assess whether a sensitisation of the trigeminovascular system persists once these pathological *noxae* have resolved, leading to headache persistence and chronification. In this context, it is worth noticing that a relevant proportion of patients without a personal history of headache had persistent headache. This fact makes us wonder whether a post-infectious aetiology may represent, in our clinical practice, an underdiagnosed cause of new daily headache. However, we are aware that to fully assess headache persistence these patients should be followed up for at least 3 months.

This is, at present, the only prospective study conducted on headache and COVID-19. We report the highest proportion of headache observed in literature (5–9), which reflects the fact that, for the first time, neurologists, working as general clinicians at the ER, could properly directly assess headache in the acute phase of a viral disease. However, we cannot estimate in our study the real prevalence of headache attributed to COVID-19 since we only included those patients attended by the investigators, who were only a part of the huge number of patients visited at the ER daily during the study period. However, it is important to point out that our results are in line with that of the European COVID-19 cohort (10), where headache and other COVID-19 symptoms were also directly assessed by clinicians through a structured questionnaire. Our strength is that the neurologists’ direct involvement made data collection on previous headache history and headache characteristics more reliable. As for the other limitations, we could not include severe COVID-19 patients due to the impossibility of conducting a full interview, or very mild patients who have gone undiagnosed and were unlikely to come to the ER, our cohort not being representative of the entire spectrum of COVID-19 patients. This fact makes it more difficult to properly assess possible associations between headache and the clinical evolution of COVID-19 in a population different from the hospitalised one. Nevertheless, a more homogeneous group makes our findings more generalisable in this specific group and useful to guide clinicians in case of future new disease peaks. Another limitation is represented by the fact that not all patients could have a confirmed diagnosis through an RT-PCR for SARS-CoV-2 due to hospital protocols, although all patients in our cohort were highly suspected cases. Moreover, we also have to consider that, as observed in a recent study, the false negative rate with RT-PCR can be high, reaching 29% (36). In addition, we did not investigate secondary causes of headache during disease evolution, making it difficult to exclusively attribute to SARS-CoV-2 possible changes in headache features during the course of COVID-19. Finally, we are also aware that the analysis of inflammatory biomarker evolution during hospitalisation counts on a small sample size. These findings should be further assessed in larger patient groups, although they represent a starting point in hypothesising and studying the link between headache, COVID-19 and IL-6.

## Conclusions

Headache associated with COVID-19 is a frequent symptom, predictive of a shorter COVID-19 duration. A relevant number of patients without a headache history have headache persisting for more than 6 weeks even when other COVID-19 symptoms resolve. Persistent headache often represents a prodromal, difficult to treat and disabling symptom of COVID-19, for which patients may seek medical attention. Efforts in the future will have to focus on better understanding its pathophysiological mechanisms, possibly involving the peripheral activation of the trigeminovascular system by inflammation or a direct role of SARS-CoV-2, in order to provide better care and new therapeutic solutions to patients.

## Clinical implications

- Headache is one of the most frequent and prominent symptoms of COVID-19, its presence being predictive of a shorter COVID-19 disease duration.
- Headache attributed to COVID-19 can have migraine-like features and in some cases can be persistent and disabling, even in patients without a previous personal headache history.
- Presence of headache, its different characteristics and evolution may reflect different pathophysiological mechanisms acting in COVID-19, making their study necessary to better understand both headache and COVID-19 itself and offer better therapeutic options.

## References

[1] Actualización no 81. Enfermedad por el coronavirus (COVID-19). 20.04. 2020 (datos consolidados a las 21:00 horas del 19.04.2020). Centro de Coordinación de Alertas y Emergencias Sanitarias. Ministerio de Sanidad. Gobierno de España, https://www.mscbs.gob.es/profesionales/saludPublica/ccayes/alertasActual/nCov-China/documentos/Actualizacion_81_COVID-19.pdf (2020, accessed 21 April 2020).

[2] Centers for Disease Control and Prevention, https://www.cdc.gov/coronavirus/2019-ncov/symptoms-testing/symptoms.html (2020, accessed 17 June 2020).

[3] Headache Classification Committee of the International Headache Society (IHS). The International Classification of Headache Disorders, 3rd edition. *Cephalalgia* 2018; 38: 1–211. 10.1177/0333102417738202 29368949

[4] De Marinis M Welch KMA. Headache associated with non-cephalic infections: Classification and mechanisms. Cephalalgia 1992; 12: 197–201. 1525796 10.1046/j.1468-2982.1992.1204197.x

[5] Mao L Jin H Wang M, et al Neurologic manifestations of hospitalized patients with coronavirus disease 2019 in Wuhan, China. JAMA Neurol 2020; 77: 683–690. 32275288 10.1001/jamaneurol.2020.1127 PMC7149362

[6] Chen N Zhou M Dong X, et al Epidemiological and clinical characteristics of 99 cases of 2019 novel coronavirus pneumonia in Wuhan, China: A descriptive study. Lancet 2020; 395: 507–513. 32007143 10.1016/S0140-6736(20)30211-7 PMC7135076

[7] Huang C Wang Y Li X, et al Clinical features of patients infected with 2019 novel coronavirus in Wuhan, China. Lancet 2020; 395: 497–506. 31986264 10.1016/S0140-6736(20)30183-5 PMC7159299

[8] Chang D Lin M Wei L, et al Epidemiologic and clinical characteristics of novel coronavirus infections involving 13 patients outside Wuhan, China. JAMA 2020; 323: 1092–1093. 32031568 10.1001/jama.2020.1623 PMC7042871

[9] Romero-Sánchez CM Díaz-Maroto I Fernández-Díaz E, et al Neurologic manifestations in hospitalized patients with COVID-19: The ALBACOVID registry. Neurology 2020; 95: e1060–e1070. 32482845 10.1212/WNL.0000000000009937 PMC7668545

[10] Lechie JR Chiesa-EstombaCM, Place S, et al Clinical and epidemiological characteristics of 1,420 European patients with mild-to-moderate coronavirus disease 2019. J Intern Med 2020; 288: 335–344. 32352202 10.1111/joim.13089 PMC7267446

[11] World Health Organization. https://www.who.int/health-topics/coronavirus#tab=tab_3 (2020, accessed 10 August 2020).

[12] Buuren S and Groothuis-Oudshoorn K. Mice: Multivariate imputation by chained equations in R. J Stat Softw 2011; 45: 3–67.

[13] Bolay H Gül A Baykan B. COVID-19 is a real headache! Headache 2020; 60: 1415–1421. 10.1111/head.13856 PMC7272895 32412101

[14] Belvis R. Headaches during COVID-19: My clinical case and review of the literature. Headache 2020; 60: 1422–1426. 10.1111/head.13841 PMC7273035 32413158

[15] Porta-Etessam J Matías-Guiu JA González-García N, et al Spectrum of headaches associated with SARS-CoV-2 infection: Study of healthcare professionals. Headache 2020; 60: 1697–1704. 10.1111/head.13902 PMC7405125 32666513

[16] Singh J Ali A. Headache as the presenting symptom in 2 patients with COVID-19 and a history of migraine: 2 case reports. Headache 2020; 60: 1773–1776. 10.1111/head.13890 PMC7300704 32521062

[17] Estudio ENE-COVID19: Primera Ronda Estudio Nacional de Sero-Epidemiología de la Infección por SARS-CoV-2 en España. Informe preliminar 13 de Mayo 2020, https://www.mscbs.gob.es/ciudadanos/ene-covid/docs/ESTUDIO_ENE-COVID19_PRIMERA_RONDA_INFORME_PRELIMINAR.pdf (2020, accessed 27 May 2020).

[18] Kaye R Chang CWD Kazahaya K, et al COVID-19 Anosmia Reporting Tool: Initial findings. Otolaryngol Head Neck Surg 2020; 163: 132–134. 32340555 10.1177/0194599820922992

[19] Beltrán-Corbellini Á Chico-García JL Martínez-Poles J, et al Acute-onset smell and taste disorders in the context of Covid-19: A pilot multicenter PCR-based case-control study. Eur J Neurol 2020; 27: 1738–1741. 10.1111/ene.14273 PMC7264557 32320508

[20] Walter EJ Hanna-Jumma S Carraretto M, et al The pathophysiological basis and consequences of fever. Crit Care 2016; 20: 200. 27411542 10.1186/s13054-016-1375-5 PMC4944485

[21] Mehta P McAuley DF Brown M, et al COVID-19: Consider cytokine storm syndromes and immunosuppression. Lancet 2020; 395: 1033–1034. 32192578 10.1016/S0140-6736(20)30628-0 PMC7270045

[22] Tanaka T Narazaki M Kishimoto T. Il-6 in inflammation, immunity, and disease. Cold Spring Harb Perspect Biol 2014; 6: a016295. 25190079 10.1101/cshperspect.a016295 PMC4176007

[23] Zhang C Wu Z Li JW, et al Cytokine release syndrome in severe COVID-19: Interleukin-6 receptor antagonist tocilizumab may be the key to reduce mortality. Int J Antimicrob Agents 2020; 55: 105954. 32234467 10.1016/j.ijantimicag.2020.105954 PMC7118634

[24] D’Alessandro A Thomas T Dzieciatkowska M, et al Serum proteomics in COVID-19 patients: Altered coagulation and complement status as a function of IL-6 level. J Proteome Res. Epub ahead of print 29 July 2020. doi: 10.1021/acs.jproteome.0c00365. 10.1021/acs.jproteome.0c00365 PMC7640953 32786691

[25] Zhou YQ Liu Z Liu ZH, et al Interleukin-6: An emerging regulator of pathological pain. J Neuroinflammation 2016; 13: 141. 27267059 10.1186/s12974-016-0607-6 PMC4897919

[26] Sarchielli P Alberti A Baldi A, et al Proinflammatory cytokines, adhesion molecules, and lymphocyte integrin expression in the internal jugular blood of migraine patients without aura assessed ictally. Headache 2006; 46: 200–207. 16492228 10.1111/j.1526-4610.2006.00337.x

[27] Yan J Melemedjian OK Price TJ, et al Sensitization of dural afferents underlies migraine-related behavior following meningeal application of interleukin-6 (IL-6). Mol Pain 2012; 8: 6. 22273495 10.1186/1744-8069-8-6 PMC3274468

[28] Ramachandran R. Neurogenic inflammation and its role in migraine. Semin Immunopathol 2018; 40: 301–314. 29568973 10.1007/s00281-018-0676-y

[29] Vaira LA Salzano G Deiana G, et al . Anosmia and ageusia: Common findings in COVID-19 patients. Laryngoscope 2020; 130: 1787. 10.1002/lary.28692 PMC7228304 32237238

[30] Li YC Bai WZ Hashikawa T. The neuroinvasive potential of SARS-CoV2 may play a role in the respiratory failure of COVID-19 patients. J Med Virol 2020; 92: 552–555. 32104915 10.1002/jmv.25728 PMC7228394

[31] Zubair AS McAlpine LS Gardin T, et al Neuropathogenesis and neurologic manifestations of the coronaviruses in the age of coronavirus disease 2019: A review. JAMA Neurol 2020; 77: 1018–1027. 10.1001/jamaneurol.2020.2065 PMC7484225 32469387

[32] Politi L Salsano E Grimaldi M. Magnetic resonance imaging alteration of the brain in a patient with coronavirus disease 2019 (COVID-19) and anosmia. JAMA Neurol 2020; 77: 1028–1029. 10.1001/jamaneurol.2020.2125 32469400

[33] Varga Z Flammer AJ Steiger P, et al Endothelial cell infection and endotheliitis in COVID-19. Lancet 2020; 395: 1417–1418. 32325026 10.1016/S0140-6736(20)30937-5 PMC7172722

[34] Hamming I Timens W Bulthuis MLC, et al Tissue distribution of ACE2 protein, the functional receptor for SARS coronavirus. A first step in understanding SARS pathogenesis. J Pathol 2004; 203: 631–637. 15141377 10.1002/path.1570 PMC7167720

[35] Baig AM Khaleeq A Ali U, et al Evidence of the COVID-19 virus targeting the CNS: Tissue distribution, host-virus interaction, and proposed neurotropic mechanisms. ACS Chem Neurosci 2020; 11: 995–998. 32167747 10.1021/acschemneuro.0c00122

[36] Woloshin S Patel N Kesselheim AS. False negative tests for SARS-CoV-2 infection – challenges and implications. N Engl J Med 2020; 383: e38. 32502334 10.1056/NEJMp2015897

